# Meaning and Wellness: A Comparative Psychobiography on Helen Suzman and Beyers Naudé

**DOI:** 10.5964/ejop.5391

**Published:** 2021-08-31

**Authors:** Carla Nel, Barbara Burnell, Paul J. P. Fouché, Roelf van Niekerk

**Affiliations:** 1 Department of Psychology, University of the Free State, Bloemfontein, South Africa; 2 Private Practice; 3 Department of Industrial and Organisational Psychology, Nelson Mandela University, Port Elizabeth, South Africa; University of Johannesburg, Johannesburg, South Africa

**Keywords:** meaning domains, holistic wellness, comparative psychobiography, eugraphic study, Beyers Naudé, Helen Suzman, anti-apartheid figures

## Abstract

This comparative psychobiographical study provides an in-depth exploration of meaning in the lives of two extraordinary individuals, Helen Suzman and Beyers Naudé. A comparison of the construction of meaning, as an important aspect of wellness within the holistic wellness model, is given for these South African anti-apartheid activists. Suzman (1917–2009) dedicated her career to opposing apartheid policy as a parliamentary politician. Naudé (1915–2004) was a renowned public figure dedicated to social justice in his role as a theologian. The holistic wellness model views the Neo-Adlerian life task of spirituality as crucial to ascribing meaning to life events, acknowledging multiple potential sources of meaning. The differences and similarities pertaining to the domains of meaning-making of these two subjects are explored. The subjects, who differed regarding biographical variables, were found to share a common sense of purpose within the same socio-political milieu. The study findings confirm that commitment to diverse sources of meaning and generativity are central to meaningfulness. This comparative psychobiographical study contributes to the eugraphic exploration of the meaning-making processes of these exemplary individuals.

Meaning making, despite being an important facet of human existence, has not been adequately explored in psychobiography (Mayer & May, 2019). The holistic wellness model views meaningfulness as important in enhancing wellness and provides a useful framework for exploring meaning-making throughout the lifespan. This model acknowledges multiple potential sources of meaning, including overcoming hardship, forming an individual life philosophy, engaging in interpersonal attachments, practising self-mortification, pursuing pleasure, striving for self-actualisation, advancing social interests or performing religious tasks (Mosak & Dreikurs, 2000; Myers, Sweeney, & Witmer, 2000). This multi-dimensional approach (Mayer, Van Niekerk, & Fouché, 2020) corresponds to the expansive and integrated model of meaning developed by Schnell (2009, 2011), in which four domains are conceptualised, consisting of 26 potential sources of meaning in life:

1. **Self-transcendence** through commitment to goals beyond personal needs, which can occur vertically (e.g., *religion* and *spirituality*) and horizontally (e.g., *social commitment*, *unison with nature*, *self*-*knowledge*, *health* and *generativity*).
2. **Self-actualisation** through the development of personal capacity by committing to sources of meaning such as *challenge*, *individualism*, *power*, *development*, *achievement*, *freedom*, *knowledge* and *creativity*.
3. **Order** by holding on to values, morals, and ethics and committing to *tradition*, *practicality*, *morality,* and *reason* as possible sources of meaning.
4. **Wellbeing and relatedness** referring to the cultivation of, and participation in, pleasurable experiences and including *community*, *fun*, *love*, *comfort*, *care*, *attentiveness,* and *harmony* as potential sources of meaning.

Within the holistic wellness model, all aspects of well-being are continuously influenced by life forces such as family, religion, education, community, media, government, and industry (Witmer & Sweeney, 1992). Furthermore, global events and issues, such as war, disease, poverty, etc., also exert an influence on well-being by influencing “the dynamics of our existential world” (Sweeney & Witmer, 1991, p. 538). Individuals’ processes of uncovering a sense of meaning differ, based on their subjective interpretations of past experiences as well as the specific, cultural realities they live and function in. Psychobiographical research can harness the value found in the comparative study of multiple lives to explore similarities and differences between individuals and their interactions with their environments, leading to a richer understanding of the subjects’ uniqueness (Isaacson, 2005). This research strives to examine how it may potentially also better illuminate a specific construct, such as meaning.

Helen Suzman (1917–2009), known for her intolerance of injustice and concern for the plight of the disenfranchised, dedicated her parliamentary career to opposing apartheid rule. Beyers Naudé (1915–2004), a renowned public figure, was dedicated to the pursuit of social justice in his role as a theologian. Previous psychobiographical studies of these subjects highlighted the centrality of a sense of meaning and purpose to their overall wellbeing (Fouché, Burnell, & Van Niekerk, 2015; Fouché, Nel, & Van Niekerk, 2017). This comparative psychobiographical study provides an in-depth exploration of differences and similarities pertaining to meaning for these two subjects.

## Method

Naudé and Suzman were selected through purposive sampling based on previous research for the eugraphic exploration of the similarities and differences in meaning-making that contribute to their exemplary life stories. These morphogenic studies (as opposed to being nomothetic or idiographic) focus on the differences and commonalities between subjects (Runyan, 1983) through serial iteration, involving side-by-side comparisons of the data sets (Isaacson, 2005).

### Data Collection

Data sources relevant to the research aims were consulted, which mainly included Suzman’s and Naudé’s autobiographical works. Other sources included published materials such as speeches and media interviews, biographical works on the subjects, the memoirs of colleagues, various published newspaper articles, as well as media interviews with, or speeches by, family, friends and colleagues. Published journal articles and unpublished postgraduate dissertations from South African university libraries were also retrieved.

### Data Extraction and Analysis

The extensive amount of data available on Suzman and Naudé needed to be reduced to a manageable amount in a systematic fashion. Firstly, the collected data was sorted according to the categories related to the meaning making model, as proposed by Schnell (2009, 2011). Secondly, Alexander’s (1988, 1990) saliency indicators were employed to reveal data most pertinent to the research topic. Data related to the domains of meaning was categorised according to the life phases of the subjects. Thereafter, the researchers were able to perform a side-by-side comparison to uncover striking and significant similarities and difference between the subjects. Additional measures to ensure data trustworthiness was ensured through prolonged, in-depth engagement with the data, obtaining feedback from key informants and triangulating data sources and analysts (Burnell, 2013; Nel, 2013). The researchers purposefully selected subjects to whom they had unbiased attitudes. Throughout the research process as more information was uncovered, the researchers took care to examine and reflect on their personal reactions towards the subjects.

### Ethical Considerations

Permission for the original psychobiographical studies (Burnell, 2013; Nel, 2013) on the two subjects was granted by the researchers’ institutional research review board, the Committee for Title Registrations of the Faculty of the Humanities at the University of the Free State (South Africa). Consent was also obtained from Suzman and the family members of Naudé. In this study, the researchers adhered to the ethical considerations pertaining to psychobiographical research (Ponterotto, 2014; Ponterotto & Reynolds, 2017).

## Findings

Six historical periods that constitute similar life phases for the subjects were identified during data collection. The findings related to the sources of meaning that featured prominently during these life phases, will be presented in the following sections.

### Phase 1: Beginnings

This phase (1915–1938) represents Naudé’s childhood and university years. His social consciousness, especially for the disenfranchised, developed and became an indelible feature of his life (Randall, 1982). It developed from his parents’ concern with broader community issues (related specifically to Afrikaners^1^1Afrikaners are descendants from European immigrants (mainly Dutch, but also French and German) who settled in the Cape Colony. A separate culture and language (*Afrikaans*) emerged (Giliomee, 2003).) and Naudé’s awareness of the devastating effects of the global Great Depression on his community (Clur, 1997). Naudé’s Christian *rebirth* at the age of 15 and subsequent commitment to his faith shaped the rest of his life (Clur, 1997). In this phase, Naudé also engaged with outdoor activities in nature and exercise (e.g., hiking), which became lifelong sources of relaxation and health (Naudé, 1995). While courting Ilse Weder (whom he later married), Naudé was exposed to a socially integrated missionary community at Genadendal, Ilse’s family home (Naudé, 1995). Due to this exposure, Naudé began to question the segregation policies of the country and his church at the time (Villa-Vicencio, 1985). While he considered himself a loyal Afrikaner, Naudé “… disliked the all-embracing control which the nationalist movement sought to exert and so shied away from the more exclusive claims of Afrikanerdom” (Clur, 1997, p. 16). Due to the books he read, as well as experiences and encounters he had at university, he “… slowly began to question the alliance to theology and nationalism, which formed the basis of his parents’ home. These were questions which, however, remained at a preliminary and latent level in his early life” (Villa-Vicencio, 1995, p. 20).

In stark contrast to that of her parents, who had fled from Jewish persecution in Russia, Suzman (neé Gavronsky) enjoyed a privileged and sheltered upbringing during this phase (1917–1936). However, young Suzman’s knowledge of their experiences sensitised her to the “evils of race discrimination” (Suzman, 1993, p. 10). Her father’s remarriage when she was 9 years old (Suzman’s mother had died shortly after childbirth) ushered in a period of community, comfort, care, and creativity for the family, with young Suzman performing at charity events hosted by her parents (Strangwayes-Booth, 1976; Suzman, 1993). Governmental policy had engineered isolation from other racial groups and placed her, even as a child, in a position of authority over the family’s Black employees, delaying the development of her later keen sense of social justice. Little information is available on religious beliefs during her childhood, as she simply stated: “we lit candles on Friday nights and had large gatherings for the Jewish festivals. My father and stepmother attended synagogue on the High Holy Days. I did not” (Suzman, 1993, p. 10). The morals, values, customs, and practices inherent to her Jewish upbringing and Catholic school education, however, influenced the formation of her identity and life philosophy, which emphasised independence, punctuality, and conscientiousness. She was a popular, sporty, competitive scholar, and performed well above average academically (Strangwayes-Booth, 1976). This phase also saw the start of a lifelong enjoyment of outdoor activities, as her relationship with nature would later support her wellness throughout her lifespan (Fouché et al., 2017). Initially enthusiastic about studying towards a postgraduate law degree, Suzman’s student years were filled with social events and international tours. Her enthusiasm and performance waned after her father refused her request to continue her economics studies in London, and she dropped out of university (Suzman, 1993).

### Phase 2: Career Initialisation

This phase (1939–1954) commenced with the completion of Naudé’s seminary training. The following year he served as a trainee minister in the Dutch Reformed Church (DRC) Wellington, Cape Province, and married Ilse. His first position as a minister in the Karoo town of Loxton made him acutely aware of the destructive impact of the segregation policies of both the church and government in the congregations he was serving (Ryan, 1990). He began to question apartheid theology and the apartheid policies of the time to a greater extent in terms of his compassion for others, a reverence for human dignity and human rights, as well as moral and ethical values (Rampen, 1972). Naudé embarked on intensive reading and self-study to allay any doubt and remedy any lacking theoretical and theological knowledge regarding the apartheid policies of the church and state (Randall, 1982). Naudé derived meaning and purpose from his community and congregational work, and the couple also enjoyed close relationships in the congregations they served. For Naudé, World War II brought to the fore issues related to the need for tolerance and reverence for human life, peace, and reconciliation (Naudé, 1995).

This phase (1937–1958) started with Suzman’s marriage at the age of 19. Her life as a housewife consisted of dinner parties, dancing, golf, and horse-riding. The rise of Nazism and World War II soon sparked a new sense of purpose for her. Suzman showed persistence in serving in the war effort—returning to university after she was turned away from the SA Women's Auxiliary Force because of her child's birth. Subsequently, she joined the War Supplies Board and performed “boring but essential” (Suzman, 1993, p. 13) duties, experiencing a shared sense of meaning with a community of Jewish South Africans. With growing awareness of the Afrikaner Nationalist inclinations of the local media and developing insight into local politics and the oppression of Black South Africans, Suzman became “utterly preoccupied” (Suzman, 1993, p. 15) with issues of racial discrimination. Helen’s ego-interests expanded significantly through her occupational role (Fouché, Nel, & Van Niekerk, 2014) as her analysis of discriminatory policies for the South African Institute for Race Relations (SAIRR) launched her political career and a commitment to oppose racial inequalities (Suzman, 1993). She demonstrated careful attention to both detail and principle and was able to consider various perspectives of an argument before formulating her own view (O’Regan, 2011). By the end of this phase, she expressed her social commitment through the demanding task of challenging apartheid as an opposition Member of Parliament (MP).

### Phase 3: Divergence

During this phase (1955–1963) Naudé worked as a DRC minister in several congregations. He visited other congregations in African, Coloured, and Indian communities, where his meeting with families made him continuously more aware of the tremendous hardship and suffering caused by segregation policies. Naudé was deeply affected by the loss of life during the Sharpeville massacre^2^2On 21 March 1960, thousands of Africans protested nationally against pass laws. A crowd protested peacefully outside the police station in Sharpeville. Police opened fire, killing 69 people and wounding 186; many shot in the back (Thompson, 2006; Tutu, 2005). (Naudé, 1995). Committed to the cause of justice and liberty (Villa-Vicencio, 1995), his sense of meaning and purpose were broadened to include not only his own community but the larger South African context. He realised the need for inter-racial association and joined a multi-racial bible study group of ministers opposed to apartheid (Randall, 1982). He also launched the Christian Institute (CI), a racially integrated organisation to expose the injustice and disruption caused by racial segregation and to support Christians who opposed this system (Kistner, 1995). In the aftermath of Sharpeville, Naudé was a delegate at a Johannesburg conference of the World Council of Churches, which was held to discuss the race situation. The conference confirmed his views that racial segregation and discrimination were immoral and biblically indefensible (Randall, 1982). Prime Minister Verwoerd (like most of White South Africa) rejected and condemned these findings and instructed all delegates to denounce them. Everyone did, barring Naudé (Villa-Vicencio, 1995). He had developed an individual life philosophy and lived out the moral and ethical values that guided his everyday life. This path, however, was irreconcilable with the mainstream Afrikaner establishment and DRC views (Ryan, 1990). Naudé subsequently resigned his position and was defrocked and stripped of the title of minister of religion. His final sermon contained the message of obedience to God and Christian beliefs over obedience to the laws of man. While Naudé remained a very traditional Afrikaner throughout his life in most respects, especially regarding gender roles, he was unable to morally reconcile himself with the political and racist views of mainstream Afrikaners, (Naudé, 1995).

During this brief period (1959–1961), Suzman observed the decolonisation of African nations as representative of ethical values related to liberty, equality, and autonomy (Suzman, 1993). The government’s continued implementation of apartheid policy had directly opposed her values and the expression of her generative concern (Fouché et al., 2014). Her growing experience of her party as an ineffective vessel for opposition culminated in a split from the United Party (Suzman, 1993). Suzman and other members of the newly formed Progressive Party endured much criticism over the retention of their parliamentary seats under a new party banner. Any moral reservations Suzman harboured, however, were dispelled in the aftermath of the Sharpeville killings, as she experienced a renewed sense of purpose: “During that tragic period, we Progs were able to show our worth” (Suzman, 1993, p. 52). Her family continued to play an integral supportive role, affirming her values and providing a sense of love, care, and comfort. Suzman also enjoyed friendship and camaraderie based on shared values in the new party. From this time onward, Helen forged a relationship with the liberal press that would provide her with opportunities for validation throughout her career (Suzman, 1993).

### Phase 4: Dissention

In this phase (1964–1976), Naudé’s full-time work at the CI focused on building inter-racial relationships. Despite encountering continued resistance and criticism and being ostracised by the majority of his beloved Afrikaner community, Naudé (1995) later reflected on the sense of meaning and purpose he derived from his attempts to raise anti-apartheid awareness among Afrikaners. Naudé worked closely with Black Consciousness Movement (BCM) leaders such as Steve Biko and Bennie Khoapa. Realising the true social, economic, and emotional impact of apartheid on the Black population, he and the CI became explicit supporters of the anti-apartheid movement (Ryan, 1990). By the end of the 1960s, the anti-apartheid movement was moving towards an armed struggle since all peaceful means had failed. While Naudé opposed an armed struggle, he did support the anti-apartheid cause and continued to assist where possible, helping young African National Congress (ANC) members to flee the country (Naudé, 1995). On 16 June 1976, Soweto school children were peacefully protesting against the use of Afrikaans as an instruction medium when police shot teargas into the crowd. The children retaliated by throwing stones, and the police opened fire, killing at least seven of the protestors. The situation escalated further, with rioting and violence erupting all over Soweto and the deployment of military and police reinforcements (Clur, 1997). Naudé urged the government to rather enter into talks with Black community leaders to end the apartheid structures, but to no avail (Naudé, 1995). Instead, the government “… resorted to enormous force to quell the protests, and by 1977, the death toll in the Black townships had risen to more than 700 …” (Ryan, 1990, p. 178).

This phase (1962–1973) saw Suzman as the sole Parliamentary representative for her party, where she endured significant burdens as she forged her lonely struggle against nationalist policy. Suzman compared aspects of the apartheid legislation to Nazi rule (Suzman, 1993) and thus viewed her work as a potentially meaningful contributor to her overall wellness (Fouché et al., 2017). She extended herself beyond parliamentary duties to attend to citizens outside the constituency she represented, including political prisoners (Suzman, 1993). As a “cheeky female with a sharp tongue which she used without regard to rank or gender” within a male- and Afrikaner-dominated Parliament (Suzman, 1993, p. 114), Suzman saw herself as an honourary representative for the oppressed and a fighter for the rights of the disenfranchised. She demonstrated her generative concern through a high level of productivity and accountability under hostile and isolating conditions (Fouché et al., 2014). Suzman’s wellbeing would have been supported by her active and socially engaged lifestyle, as well as the care within her family, despite occasional political disagreements. As her work began to attract international attention, a kind of personality cult formed within the anti-apartheid press and among voters, resulting in a widespread view of her as the unofficial party leader (Nel, 2013; Strangwayes-Booth, 1976).

### Phase 5: Overt Opposition

Naudé continued to speak out against the apartheid system during the period 1977–1987, as the Black population grew increasingly discontented and resistant, cautioning that “… the institutionalised brutality of the apartheid system had engendered so much bitterness and hatred that many Black leaders were coming to the conclusion that counter-violence was a tragic inevitability” (Ryan, 1990, p. 184). He continued to engage with some Afrikaners who had also begun questioning the apartheid system (Ryan, 1990). By October 1977, the CI had been closed down by the government, with Naudé and the CI management receiving banning orders. Despite constant surveillance and strict restrictions, Naudé found ways to continue assisting with anti-apartheid activities (Naudé, 1995). Through his involvement with ANC activities, he hoped to contribute to a broader understanding of what the ANC was advocating for and hoped it would minimise the armed struggle (Villa-Vicencio, 1995). This reflected Naudé’s reverence for human life, compassion, and service to others. He attempted to serve both the disenfranchised Black population and educate and enlighten the White (specifically the Afrikaner) population (Naudé, 1995). Though stressful and challenging, this phase proved to be a spiritually rich and meaningful period for Naudé. His strong conviction regarding his actions, as well as his connection with a larger community, sustained and enriched him (Naudé, & Sölle, 1986). Naudé saw reconciliation between races as part of his spiritual duty and remained in service to others. He emerged as an important church leader after the banning order and was elected as General Secretary of the South African Council of Churches in 1985 (Tutu, 2005). In this position, he addressed communities, visited the loved ones of victims of violence related to anti-apartheid demonstrations, and continued to participate with other clergies in protest marches against apartheid (Ryan, 1990). In a sense, there was also some reward for the long years of suffering for his beliefs that apartheid was unbiblical, unethical, and morally indefensible (Tutu, 2005).

At the start of this phase (1974–1988), greater electoral success ensured that Suzman was no longer serving as the party’s sole MP. She maintained exemplary productivity levels but enjoyed the newfound camaraderie of working towards shared goals in a more supportive environment. Generativity was again expressed through her commitment, involvement, and concern for the community at large, she consulted with local leaders and opposed forced removals throughout the country (Fouché et al., 2014). She continued to enjoy the support of her family, a sense of connectedness with a like-minded community of individuals, and the validation of her work. However, Suzman also faced conflict and controversy related to her unpopular opposition of economic and cultural sanctions instituted against South Africa at the time. Not known to compromise on her sense of justice for the sake of interpersonal harmony, Suzman remained undeterred, maintaining that she based her opinions on practicality and reason (Suzman, 1993).

### Phase 6: Final Phase

This phase (1988–2004) coincides with the abolishment of the apartheid system and the birth of a new dispensation and democratic society. Naudé was involved in the political talks as part of the ANC delegation (Ryan, 1990). He had always believed that negotiation between the government and the ANC would be the only way to broker a peaceful resolution for the problems in South Africa (Potgieter, 1994) and was able to experience the beginning of the equality all the anti-apartheid activists had worked towards (Naudé, 1995). In 1994, the DRC made an apology to Naudé and Ilse. Being able to return to the DRC and, to an extent, to the Afrikaner community was deeply meaningful to him (Tutu, 2005). Into his 80s, Naudé continued to work in service of others and play a part in South Africa’s public scene. The ideal of justice, equality, and the hope of a unified South Africa for future generations remained an ideal for which Naudé strove to the end of his life (Clarke, 2004).

This phase (1989–2009) started with Suzman’s retirement, where after her social interest and generative commitment did not seem to diminish, sustained by continued efforts to advance human rights and civil liberties for future generations (Fouché et al., 2014). Intensely frustrated by her decrease in mobility as she aged, Suzman reflected favourably in her memoirs on her contributions to the abolition of apartheid (Suzman, 1993). The post-apartheid media was a continued presence as they sought out her opinion and comment, validating her continued presence in the country’s political landscape. She received various accolades and honours during her retirement, locally and abroad, which would have undoubtedly facilitated a sense of meaning and purpose regarding her life’s work (Nel, 2013). In the last phase of her life, her values were not only shared by her family, friends, and community but were mirrored in the ideals enshrined in the new Constitution (Eglin, 2007).

**Figure 1:**
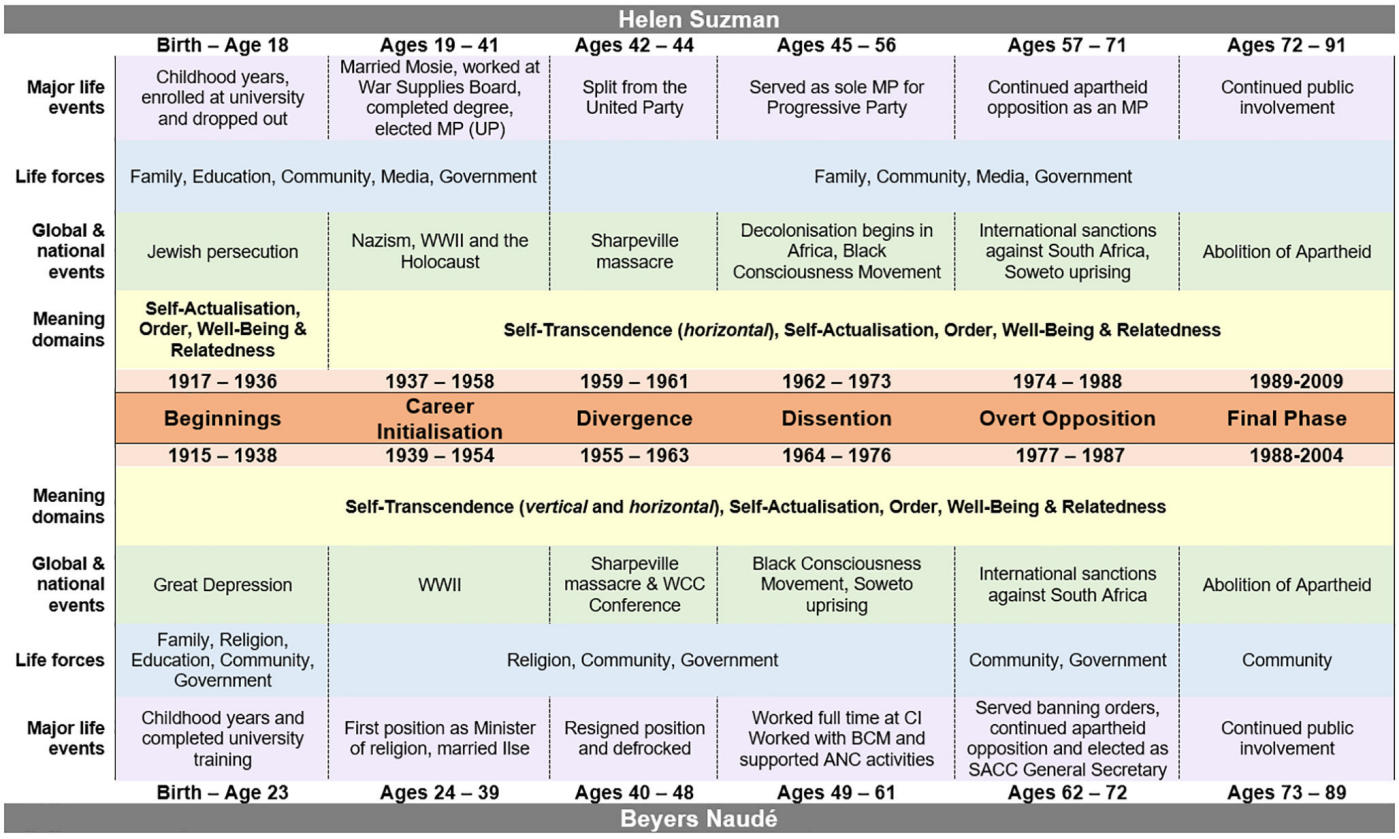
A Comparative Biographical Chart for Suzman and Naudé

To illuminate important similarities and differences in their cultivation of a meaningful, fulfilling life narrative, the comparison of the subjects in terms of the domains of meaning, life forces and global events are summarised in Figure 1.

While the subjects shared a common sense of purpose, they differed on significant variables, such as gender, language, culture, religion, and economic status. In the following discussion, the similarities and differences between the subjects related to domains of meaning are highlighted.

## Discussion

Naudé and Suzman both reflected on their lives and their opposition to the apartheid system and the sense of purpose and meaning derived from it. Suzman (1993) conveyed the sense of purpose obtained through her career: “I think it was a tremendous privilege to have been in Parliament, to have played a part in keeping values alive which most of us believe in, to have allowed nothing to go by default, to have had eyeball-to-eyeball confrontations with the government about all its offensive actions, and especially to have been able to intercede on behalf of victims of apartheid…” (p. 272). Naudé reflected on his life’s work: "What is of importance to you is your experience of life, of an inner peace, of a strength of faith, of a continuation of your commitment… you simply do not regard the traditional value systems… meaningful any longer…these values lost their meaning for me, and therefore an inner peace of mind came, also a loss of fear, that even if somebody asked me I’d say, well, suppose you go back, suppose now that you are being threatened, suppose that you may lose your life tomorrow, then, well, my response is, well if that happens, so what? Isn’t then the death which you experience as a result of what you try to be in the deepest sense of the word, isn’t that then something in a certain sense of a crowning of your whole life and what you try to convey?" (Naudé & Sölle, 1986, pp. 23–24).

### Domain: Self-Transcendence

This domain would be the most defining characteristic in the life stories of both Suzman and Naudé. Regarding horizontal self-transcendence, *generativity* and *social commitment* first became evident for Suzman during her second life phase with her involvement in the war effort and local politics. Throughout subsequent life phases, Suzman demonstrated a significant commitment to these sources of meaning. These were also the most prominent horizontal sources of meaning that featured in the data on Naudé, for whom social commitment was already a prominent aspect from the first life phase. Both subjects recalled the impact of their families’ demonstrations of concern for their respective communities. Furthermore, both subjects had expanded on their parents' commitments by developing their own ideas around social commitment and generativity to include the larger, multi-racial society of South Africa. For both Naudé and Suzman, increased knowledge regarding the full negative impact of racial rule sparked a critical re-evaluation of the policies of government (as well as, for Naudé, a questioning of the segregation policies of the church). Throughout their lives, both demonstrated generative concern through their work in general and, specifically, through their commitment to those groups most adversely affected by the apartheid system. Both subjects were driven to active opposition of the apartheid system by their concern for the wellbeing of future generations. Both Suzman and Naudé demonstrated lifelong commitments to other horizontal sources of self-transcendence, such as *unison with nature* and the development of *self-knowledge.* For both subjects, the experience of good *health* as a potential source of meaning was only challenged during the last phase of life as advancing age gradually led to declining health and mobility.

Some individual differences in self-transcendence were also observed. Naudé’s experience of this domain was significantly influenced by commitments to vertical sources of self-transcendence through religious and spiritual experiences. Starting from Naudé’s strict, religious upbringing and his “Christian rebirth” in the first life phase, this domain continued to feature prominently throughout subsequent life phases, as his commitment to his faith continued to guide and inform many of his decisions and actions. In contrast, the data on Suzman’s life placed little emphasis on *religion* or *spirituality* as sources of meaning in her life, with her experience of self-transcendence, therefore, most prominent on the horizontal plane.

### Domain: Self-Actualisation

Naudé and Suzman both demonstrated a high degree of committed striving towards self-actualisation throughout their lives. There were, additionally, several similarities in the potential sources of meaning within this domain prominent in the historical data on both subjects. From the second phase onward, Naudé demonstrated commitment to *development* (personal growth), *knowledge* and *individualism* (autonomy and independence) as he embarked on a process of self-study and development to answer certain questions regarding apartheid theology for himself. This put him on a course toward openly challenging and opposing the apartheid government and policies as he developed his individual ideas and beliefs. In terms of these domains, Suzman demonstrated a prominent commitment to *development* and *knowledge, achievement*, *challenge* and *individualism*. Since her first life phase, she took pride in developing her sense of determination and conscientiousness. She was highly competitive throughout her life, later enduring several personal and professional challenges in performing her duties as an MP autonomously. Her meticulous acquisition of information and thorough consideration of multiple viewpoints formed the foundation of her political and economic arguments.

### Domain: Order

Data on both subjects' lives contained numerous instances of commitment to sources of meaning within this domain. Suzman, for whom the domain of order was the most prominent of the four meaning domains throughout her adult life, demonstrated her characteristic commitment to *practicality*, *morality,* and *reason* through her mastery of detail and attention to principle throughout her career. Naudé became increasingly concerned for the wellbeing of future generations of South Africa should the government not change course. To this end, he warned of the unfeasibility and unsustainability of the apartheid system, demonstrating his commitment to *practicality* and *reason*. An overlap was, therefore, observed between sources such as morality in the domain of order and sources from other dimensions, such as self-transcendence (particularly generativity and religion). For example, Naudé lived out his *religious* beliefs in ways that relate closely to *morality*. Similarly, Suzman’s sense of morality in her fight for justice for apartheid victims was expressed through well-reasoned arguments of economic and political practicality, coupled with her generative concern towards others and future generations.

One difference between the subjects emerged in this domain. *Tradition* featured less prominently in the evidence on Suzman, as she demonstrated little commitment to the conservation of traditional and cultural roles and values. Conversely, Naudé described himself as a very typical, traditional Afrikaner man despite his anti-apartheid views.

### Domain: Wellbeing and Relatedness

Naudé and Suzman both demonstrated a high degree of commitment to sources of meaning related to this domain. For both subjects, a sense of *care* and *community* featured prominently throughout their work. From the first life phase onward, Suzman enjoyed and cultivated family relationships, close friendships, and social interactions characterised by *fun*, *love*, *comfort*, *care* and *attentiveness.* During the final phase of her life, themes of relatedness feature prominently in her reflections on her life and her work. Similarly, Naudé forged close friendships that were also a source of *fun* for him, and his family was a continued focus of his *love*, *comfort*, *care* and *attentiveness.* While he experienced rejection from the majority of his Afrikaner community, Naudé continued to engage with fellow Afrikaners to influence their beliefs and convince them of his anti-apartheid views and forged relationships with different communities and groups, such as with young Black activists. Naudé found his work with families of victims of the violence and his own participation in the anti-apartheid protests and demonstrations as particularly meaningful. In the last phase of his life, the public apology by the DRC and the welcoming back into the “fold” of the Afrikaner community that it signified was also very meaningful to Naudé. Similarly, Suzman’s activities within different communities also served as an outlet for her sense of generativity, again highlighting the potential overlap between different domains of meaning.

A key difference between the subjects regarding this domain involves *harmony* as a source of meaning. It featured very prominently in Naudé’s life, since he was deeply affected by violence, intolerance and the lack of reverence for human life displayed during WWII, the Sharpeville massacre and Soweto uprising. He advocated for tolerance and reconciliation between races in South Africa. During the last phase of his life, Naudé found particular meaning and purpose in contributing to the post-apartheid South African society and a new, harmonious, equitable dispensation for all races and it continued to be the driving force for Naudé as he continued his public activities in post-apartheid South Africa. Suzman, on the other hand, seemed to have been driven more by her desire to promote the principle of justice than a striving for harmony, with the domain of order, thereby influencing her approach to matters of community and relatedness. In trying to protect the vulnerable against harm, she was quick to voice disagreement, often did so harshly, and she easily engaged in debate, even with those closest to her. She did, however, demonstrate a high degree of commitment to *harmony with the self* through congruence between actions and values. Her utilisation of relatedness as a domain of meaning, therefore, was seemingly governed more by aspects such as love, care and attentiveness than striving towards interpersonal harmony.

### Conclusions

The findings of this study suggest the importance of commitment to diverse sources of meaning, as well as the centrality of self-transcendence and generativity, to the experience of meaningfulness. This echoes previous findings: firstly, that a strong sense of purpose and meaning in life is most likely to be achieved in the presence of commitments and intentional investments to numerous and diverse potential sources of meaning (Ryff & Singer, 1998; Schnell, 2011) and secondly, that generativity as a source within the domain of self-transcendence, is the best predictor of meaningfulness (Schnell, 2011). The data of both Suzman and Naudé emphasise the interconnectedness of the four domains of meaning, with potential overlap observed in how behaviours may relate to more than one domain. This indicates the lifelong diversity of sources of meaning in the lives of these subjects, as well as the interconnectedness of sources of meaning in the lives of individuals.

Life forces and global events, as well as the subjects’ reactions towards them, were found to significantly contribute to the sources of meaning available to the subjects and seemed to influence the domains of meaning prominent in their lives at any given stage. It seemed that for both subjects, supportive life forces facilitated their wellness and ability to experience meaningfulness, while their reaction to adversities and challenges provided them with opportunities for deepening their sense of purpose. This finding affirms the potential influence of these factors on individual wellness, as conceptualised by the holistic wellness model (Myers et al., 2000).

This study demonstrated how the domains and sources of meaning as conceptualised by Schnell (2009, 2011) could span across all the life tasks contained in the holistic wellness model (Myers et al., 2000), thus suggesting a potential application of these sources and domains of meaning as indicators of overall wellness. The authors suggest that future research projects focusing on this area may uncover further correlation or integration between meaning in life and holistic wellness.
